# Flexible Chitosan-Based Capacitive Humidity Sensors for Respiratory Monitoring

**DOI:** 10.3390/s24051352

**Published:** 2024-02-20

**Authors:** Miaoxin Pan, Jumei Zhou, Shichen Weng, Xingjian Wu

**Affiliations:** School of Maritime and Transportation, Ningbo University, Ningbo 315800, China; 2111084071@nbu.edu.cn (M.P.); wengshichen@nimte.ac.cn (S.W.); 216003592@nbu.edu.cn (X.W.)

**Keywords:** flexible humidity sensor, respiratory monitoring and decoding, chitosan

## Abstract

As one of the most important human health indicators, respiratory status is an important basis for the diagnosis of many diseases. However, the high cost of respiratory monitoring makes its use uncommon. This study introduces a low-cost, wearable, flexible humidity sensor for respiratory monitoring. Solution-processed chitosan (CS) placed on a polyethylene terephthalate substrate was used as the sensing layer. An Arduino circuit board was used to read humidity-sensitive voltage changes. The CS-based sensor demonstrated capacitive humidity sensitivity, whereby the capacitance instantly increased from 10^−2^ to 30 nF when the environmental humidity changed from 43% to 97%. The capacitance logarithm sensitivity and response voltage change was 35.9 pF/%RH and 0.8 V in the RH range from 56% to 97%. And the voltage variation between inhalation and exhalation was ~0.5 V during normal breathing. A rapid response time of ~0.7 s and a recovery time of ~2 s were achieved during respiration testing. Breathing modes (i.e., normal breathing, rest breathing, deep breathing, and fast breathing) and tonal changes during speech could be clearly distinguished. Therefore, such sensors provide a means for economical and convenient wearable respiratory monitoring, and they have the potential to be used for daily health examinations and professional medical diagnoses.

## 1. Introduction

Respiratory monitoring plays an important role in the diagnosis of many cardiovascular and mental diseases [1]. By monitoring and analyzing the physical movement of the chest or abdomen, contact monitoring equipment can reflect respiratory status [2]. Noncontact monitoring equipment captures the inspiratory and expiratory behavior of the abdomen or chest using visual perception technology [3], senses temperature changes in mouth and nose breathing using infrared thermal imaging [4], and senses humidity changes in mouth and nose breathing using sensors [5]. Noncontact monitoring is a popular method because it reduces the discomfort and environmental interference of wearable devices.

Recently, flexible humidity sensors have been widely researched for respiratory monitoring [6,7]. Generally, a high-performance humidity sensor for respiratory monitoring should have high sensitivity, fast response and recovery speeds, a wide monitoring range, good stability, and low fabrication cost, and it should be simple [8]. Liang et al. reported a stretchable polydopamine-based hydrogel humidity sensor that exhibited very high (13,462.1%/% relative humidity (RH)) sensitivity and demonstrated good real-time respiratory monitoring [8]. To simultaneously satisfy high sensitivity and fast response/recovery speed, Song et al. presented a paper-based capacitive humidity sensor that used graphene oxide (GO) as a moisture-sensitive material. It achieved an ultrahigh sensitivity of 8504% at 99% RH and had a fast response/recovery time (170/40 s) at an operating voltage of 500 Hz [9].

Common humidity sensors include resistors [10], capacitors [11], acoustic waves [12], optical fibers [13], quartz crystal microbalances (QCMs) [14], and field effect transistors [15]. Of these, the capacitor humidity sensor has become the most common humidity sensor in the market because of its advantages of low power consumption, low cost, and high efficiency. Currently, the most common humidity-sensitive materials used to make humidity sensors are polymers [16] and ceramic materials [17]. Chitosan (CS) is a natural semicrystalline polysaccharide biopolymer mainly derived from chitin, which is the second richest natural polymer on earth after cellulose. It exhibits hydrophilicity, biocompatibility, chemical inertia, nontoxic and antibacterial properties, and excellent film-forming ability [18]. CS/GO/SnO_2_ humidity sensors exhibit high responsiveness, high sensitivity (402.5 kΩ/%RH), and rapid response and recovery times (8 s/8 s) at high RH levels [19]. CS-based humidity sensors can be used to monitor human health [20]. CS-based humidity sensors have been proposed as impedance-type humidity sensors.

In this study, a flexible capacitive humidity sensor using CS on a polyethylene terephthalate (PET) substrate as the sensing layer is introduced. The sensor was fabricated using a low-cost solution and spraying process. The capacitance and impedance characteristics of the sensor under different humidity environments were investigated. An Arduino circuit board was used to convert humidity-sensitive capacitance changes into voltage changes. An experiment demonstrated that the readout voltages were very sensitive to humidity and respiratory status. The sensor can clearly identify four different breathing modes: normal breathing, deep breathing, rest breathing, and fast breathing. It can also simultaneously clearly identify tonal changes when the wearer is speaking, and it has the potential for information transmission under special circumstances. Such flexible CS-based sensors can provide an economical and convenient solution for people who require respiratory monitoring.

## 2. Materials and Methods

### 2.1. Flexible Interdigital Electrode Substrate Preparation

**Figure 1:**
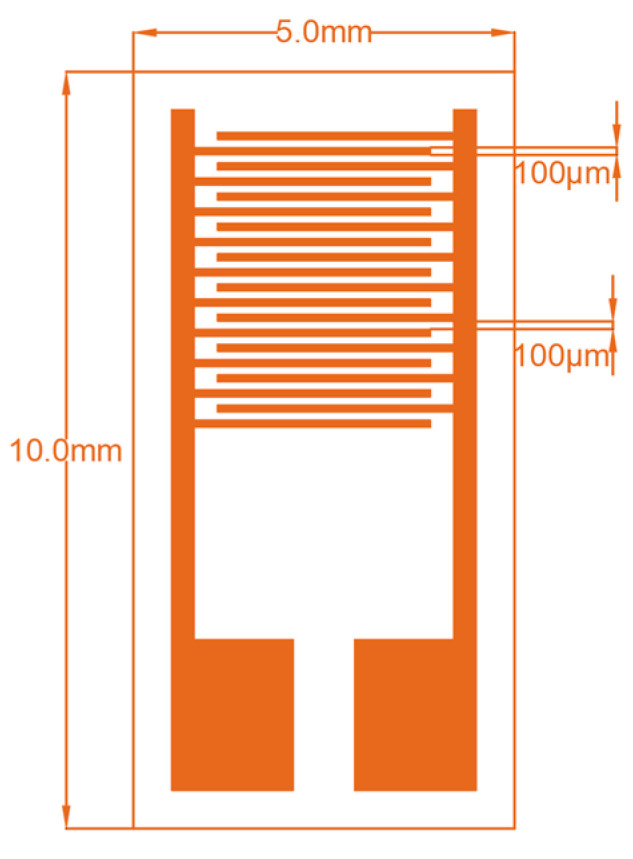
IDE structure with dimensions.

Flexible PET was used as the substrate to fabricate the humidity sensor. The interdigital electrodes (IDEs) were deposited on the PET substrate. The flexible IDE substrates used in this study were commercially purchased from Huizhou Xinwenxiong Commerce & Trade Co., Ltd., Huizhou, China. The structure and dimensions of the IDE substrate are shown in Figure 1. It has 10 IDE finger pairs. The finger width is 100 μm, the finger spacing is 100 μm, and the finger length is 3.3 mm. The Cu/Ni/Au electrode thin films are 12, 4, and 1 μm thick, respectively. The IDE substrate was ultrasonically cleaned for 20 min in purified water and then dried.

### 2.2. Preparation of Chitosan-Based Sensor

**Figure 2:**
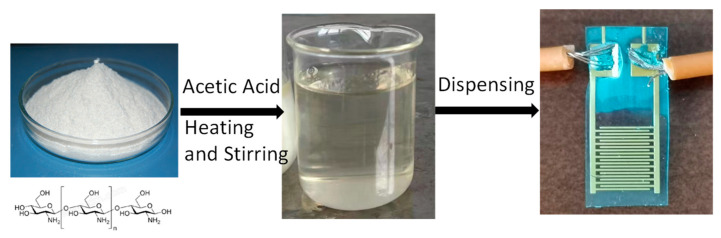
Fabrication of the chitosan-based humidity sensor.

Figure 2 depicts the fabrication process of the CS-based sensors. CS powder was used to prepare the sensitive film for the humidity sensor. First, CS (1.5 g) was dissolved in acetic acid (2% vol. 100 mL) at 60 °C and stirred for 4 h until completely dissolved. A transparent, light-yellow CS solution was obtained. Then, the CS solution was sprayed onto the surface of the IDEs and left to dry at room temperature for 24 h. Finally, connection wires were soldered to the IDEs.

### 2.3. Experimental Setup

To provide various stable RHs, sealed bottles of saturated salt solutions were prepared to simulate different RH environments: K_2_CO_3_ (43% RH), NaCl (75% RH), KCl (84% RH), and K_2_SO_4_ (97% RH). The capacitance and resistance of the humidity sensor were measured using a digital multimeter sensor placed into the sealed bottles. The complex impedance characteristics of the CS films were also investigated using an impedance analyzer (Solartron 1260). The measured humidity ranged from 43% to 97% RH. All the humidity tests were performed at room temperature (25 °C).

**Figure 3:**
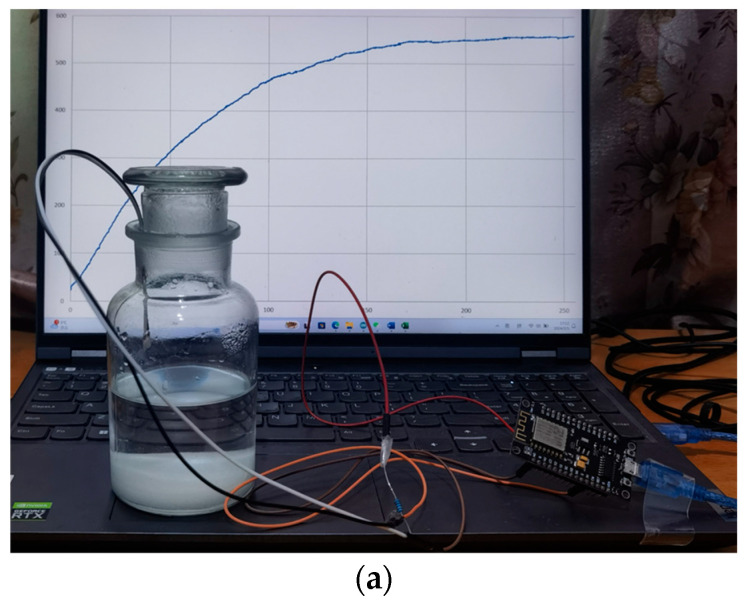

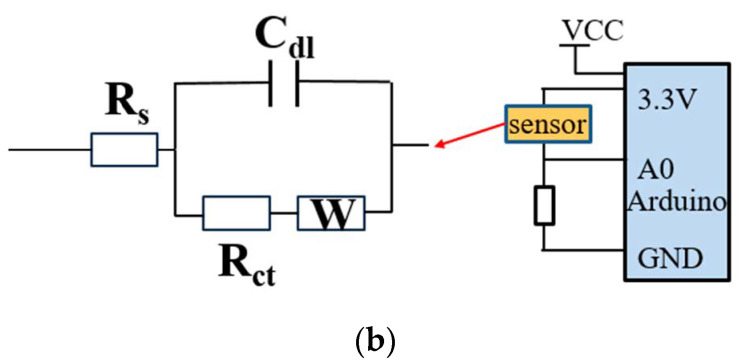
(**a**) The humidity testing and respiratory monitoring system with the Arduino circuit board. (**b**) Testing equivalent circuit and equivalent circuit model for sensors.

The humidity testing and respiratory monitoring system comprised the development board, the Arduino program, and the humidity sensors. Figure 3a shows that the humidity sensor was connected to the internal circuit of the development board, with the sensor in the sealed bottle. The equivalent circuit is shown in Figure 3b. The CS-based sensor is represented by the left circuit model, including a double-layer capacitance (C_dl_), a charge transfer impedance (R_ct_), a Warburg impedance (W), and a solution impedance (R_s_). The Arduino circuit board converted the humidity-sensitive capacitance changes into voltage changes. The Arduino circuit board read the voltage from analog signal port A0 and transmitted it to the upper computer via a USB. Capacitance changes caused by humidity can affect variations in the partial readout voltage.

## 3. Results and Discussion

### 3.1. Capacitive Humidity Sensor

**Figure 4:**
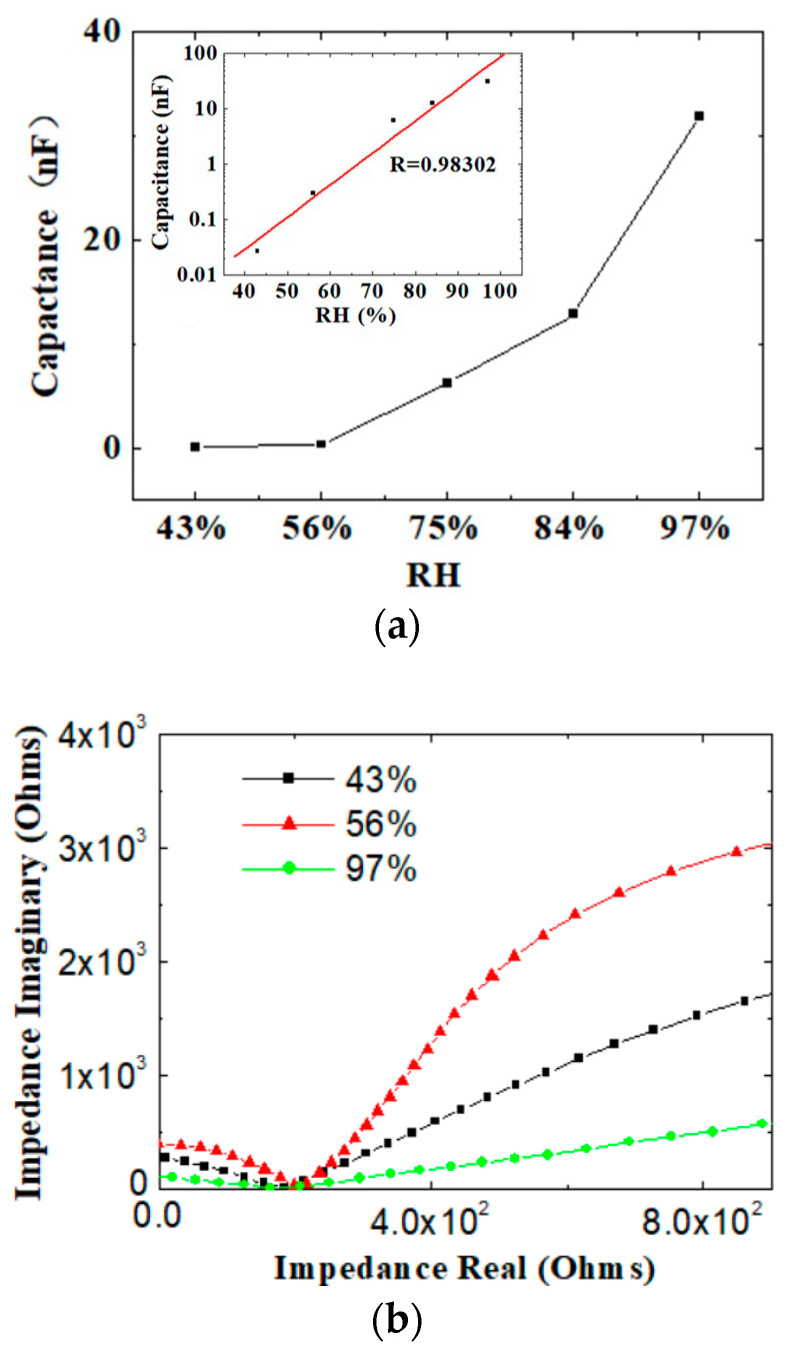
(**a**) Capacitance of the sensor as a function of the different humidities, inset: capacitance–RH with logarithmic vertical axis. (**b**) Impedance characteristics of the sensor at 43%, 56%, and 97% humidity.

The CS-based sensors were demonstrated to be sensitive to environmental humidity. The humidity of the ambient air was 56% at 25 °C (i.e., room temperature). The sensors were placed in the sealed bottles above the saturated salt solutions. The experimental results showed that the capacitance between the two electrodes of the humidity sensor changed as the environmental humidity changed. Figure 4a shows that the capacitance was very low, about 10^−2^ nF at a low humidity (~43% RH), and it increased to ~0.3 nF at 56% RH. It started to increase significantly after 75% RH and reached a maximum of ~30 nF at 97% RH. It exhibited a 100-fold change in capacitance in the high-humidity range between 56% and 97%rH, comparing with a 75-fold change in resistance between 20% and 90% RH in the chitosan humidity sensor on the polyurethane foil [21]. The fitting curve (inset) exhibited linearity with R = 0.983 when the capacitance and relative humidity was plotted with a logarithmic vertical axis. Referring to [22], we calculated the logarithm sensitivity by using

(1) $$S=\frac{\log(C_{fin}-C_{ini})}{{RH}_{fin}-{RH}_{ini}}$$

where *C*_fin_ is the capacitance at a specific humidity and *C*_ini_ is the initial capacitance, *RH*_fin_ and *RH*_ini_ are the correspongding relative humidities, and a sensitivity of 35.9 pF/%RH in the RH range of 56–97% was obtained. Comparing with flexible humidity sensors fabricated through solution processing in the literature [22], our sensors exhibited a relatively high sensing performance. However, the experiment indicated that the humidity-sensitive resistance changed very little. Therefore, the CS-based humidity sensor was a type of capacitive humidity sensor.

The impedance characteristic curves under different humidity environments were also investigated. Figure 4b shows that all the impedance spectrums plotted as a semicircle in the high- and middle-frequency regions and as a straight line in the low-frequency region. The intersection points of the semicircles on the impedance real axis were similar, indicating that charge transfer impedance in different humidity environments was similar. However, the slopes of the straight lines differed at different humidities (i.e., 43%, 56%, and 97%). The capacitive reactance was greater at low humidity and smaller at high humidity. According to the classical Nyquist equivalent circuit, the semicircular line region reflected the charge transfer resistance between the CS film and the IDE, while the straight line reflected the Warburg impedance of the ion diffusion in the CS film. In a humid environment, water molecules are adsorbed onto the surface of the humidity-sensitive layer of CS, and hydrogen bonds are formed between the oxygen in the water molecules and the hydrogen in the amino group of the CS. Chemical adsorption occurs through hydrogen bonding, resulting in the formation of multiple layers of water. This series of water layers accelerates the transfer rate of water molecules and ions, and ion transfer becomes the main conduction mode of this process in low-frequency regions.

**Figure 5:**
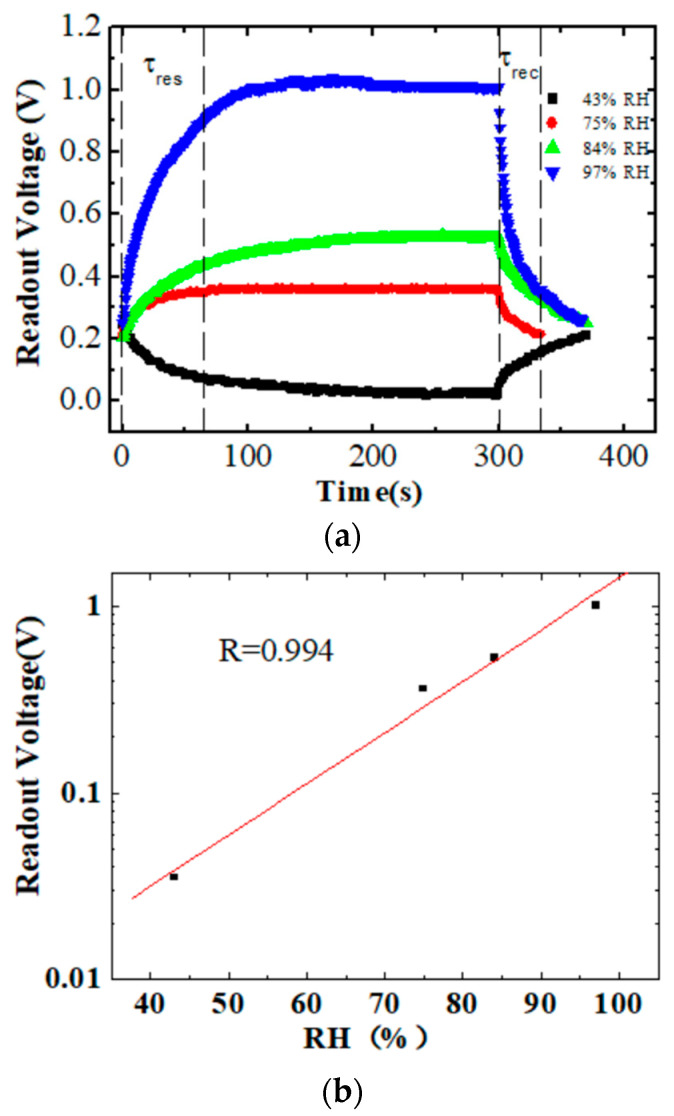
(**a**) Readout voltage from analog signal port A0 when the humidity sensor was placed in different humidity environments. (**b**) Readout voltage of the sensor as a function of the different humidities with logarithmic vertical axis.

Different humidity responses were tested. The readout voltage from A0 was detected after the humidity sensor was placed from ambient air into the sealed bottles above the saturated salt solutions. As Figure 5 shows, the readout voltage increased to a steady platform of ~1.01, 0.53, and 0.36 V for 97% RH, 84% RH, and 75% RH, respectively, which represent humidities greater than ambient air humidity. However, it decreased to a steady platform of ~0.03 V for 43% RH, which represented humidity lower than ambient air humidity. According to the equivalent circuit shown in Figure 3b, at ambient air humidity, the total reactance of the sensor divided a high voltage level, and the initial voltage from A0 was about 0.2 V. As the RH increased above ambient air humidity, the total reactance decreased. As a result, the voltage drop of the sensors decreased, and the readout voltage from A0 increased. The reason for this is the opposite of that at 43% RH. The response time (τ_res_) and recovery time (τ_rec_) were defined as the time taken for the readout voltage to reach 90% of the total voltage change. The response time and recovery time were approximately 68 and 42 s, respectively, for the humidity range from 56% RH to 97% RH. However, the times were slower for a lower RH. This indicates that, in high humidity, water molecules are easily and rapidly adsorbed onto the surface of CS, which accelerates ion conduction and capacitance changes. In Figure 5b, the readout voltage vs. RH is plotted with a logarithmic vertical axis. It exhibited good fitting linearity with R = 0.994 in the high-humidity range. As an RH range of 43–97% is dominant during exhaled breath, a good sensing performance of the chitosan-based humidity sensor in the RH range of 43–97% is favorable for monitoring human respiration.

### 3.2. Respiratory Monitoring System Testing

**Figure 6:**
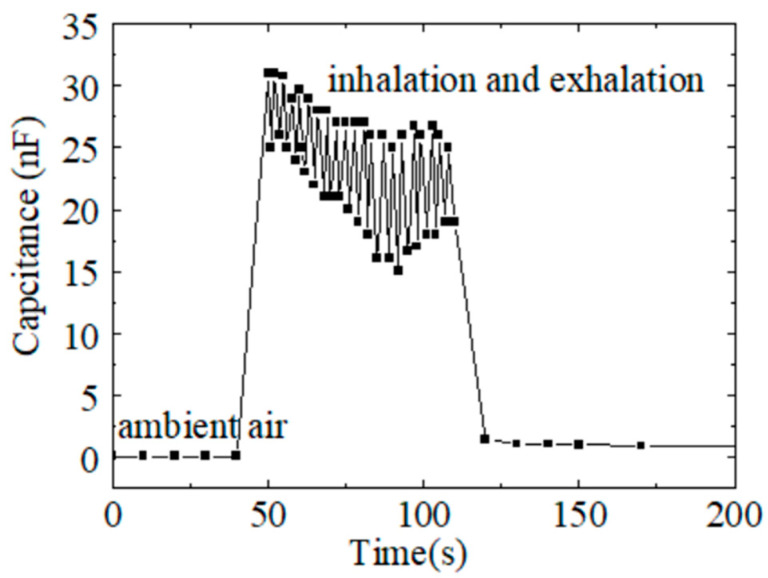
Capacitance variation in the humidity sensor during mouth breathing.

The humidity of air exhaled from the human body is close to saturation; however, the concentration of water molecules during inhalation and exhalation shifts. Thus, CS-based humidity sensors can be placed under the mouth or nose to monitor human breathing. Figure 6 shows the capacitance change in the sensors during mouth breathing. The initial capacitance was low in ambient air. The capacitance increased to ~30 nF when detecting exhaled air flow. The breathing sensitivity, S, was around 10,000%, calculated as follows [9]:

S (RH) = C_RH_/C_0_ × 100%, (2)

where C_RH_ is the capacitance at the saturation humidity of exhaled air, and C_0_ is the capacitance at the humidity of ambient air. The C_RH_ oscillates during inhalation and exhalation. Since the respiratory rate can be obtained by the response peak, and the complete response and recovery curve of the humidity sensor is not necessary [23], our CS-based humidity sensors with a high sensitivity and relatively fast response and recovery times are suitable for monitoring human breathing.

**Figure 7:**
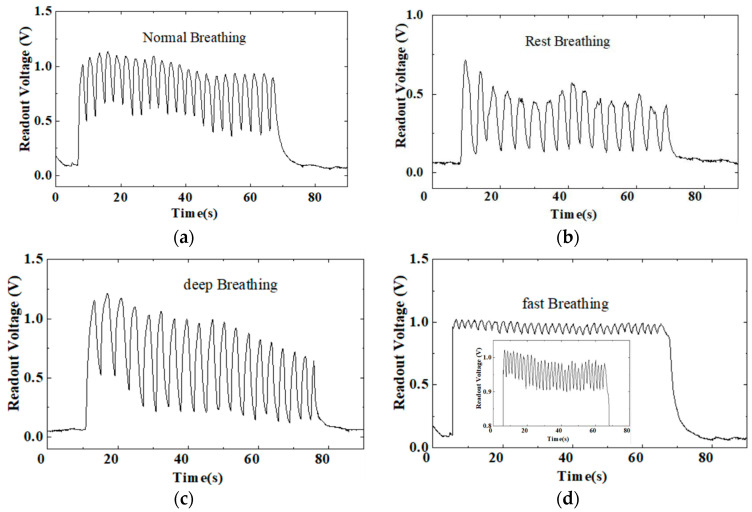
Humidity sensor response to different mouth breathing patterns: (**a**) normal breathing; (**b**) rest breathing; (**c**) deep breathing; and (**d**) fast breathing.

Figure 7 shows the readout voltage from A0 of different breathing modes when the sensor was placed under the subjects’ mouth. The measurement protocols were schemed as follows: the subject was in the state of normal breathing during testing, defined as “normal breathing”; deep breathing deliberately, defined as “deep breathing”; resting state with eyes closed during testing, defined as “rest breathing”; and rapid breathing deliberately, defined as “fast breathing”. The readout voltage was around 0.1–0.2 V in ambient air. During normal breathing (Figure 7a), the readout voltage instantly changed up to ~1.0 V during the exhalation moment and was reduced to ~0.5 V during inhalation. It then oscillated between 0.5 and 1.0 V. The voltage variation between inhalation and exhalation was ~0.5 V, with a variation trend in accordance with the capacitance variation shown in Figure 6. The mechanism can be explained as follows: Initially, the capacitive humidity sensor is charged at an RH of 56% in ambient air. Then, when the sensor is placed in the saturation humidity of the exhaled air, the capacitive sensor begins discharging as the sensor’s capacitance increases. Therefore, the humidity sensor voltage gradually decreases, while the readout voltage increases. The Arduino circuit with the CS sensor system converts humidity-induced sensor capacitance changes into voltage changes, which can be used to clearly visually monitor respiration status. In Figure 5a, we obtained a readout voltage platform of ~0.2 V and ~1.01 V for 56% RH and 97% RH, respectively. Here, we calculated the response time and recovery times during respiration testing for readout voltage changing from 0.2 V to 1.0 V. The response and recovery times were approximately 0.7 and 2 s, respectively. These evaluated response and recovery times meet the reference values for respiration detection. We assumed that this fast response time for the large dynamic change is favorable for distinguishing the respiration intensity and frequency of different breathing modes. Figure 7b shows that the peak voltage for exhalation was smaller, about 0.55 V, indicating the respiration intensity is lower in the rest breathing mode. A large voltage variation of ~0.75 V indicated the deep breathing mode while a small voltage variation of ~0.15 V indicated the fast breathing mode. By counting the regularly fluctuating changes in 1 min, different respiration frequencies of 16, 18, 20, and 32 breaths per minute (bmp) were obtained for rest, deep, normal, and fast breathing, respectively. 

### 3.3. Speech Recognition Testing

**Figure 8:**
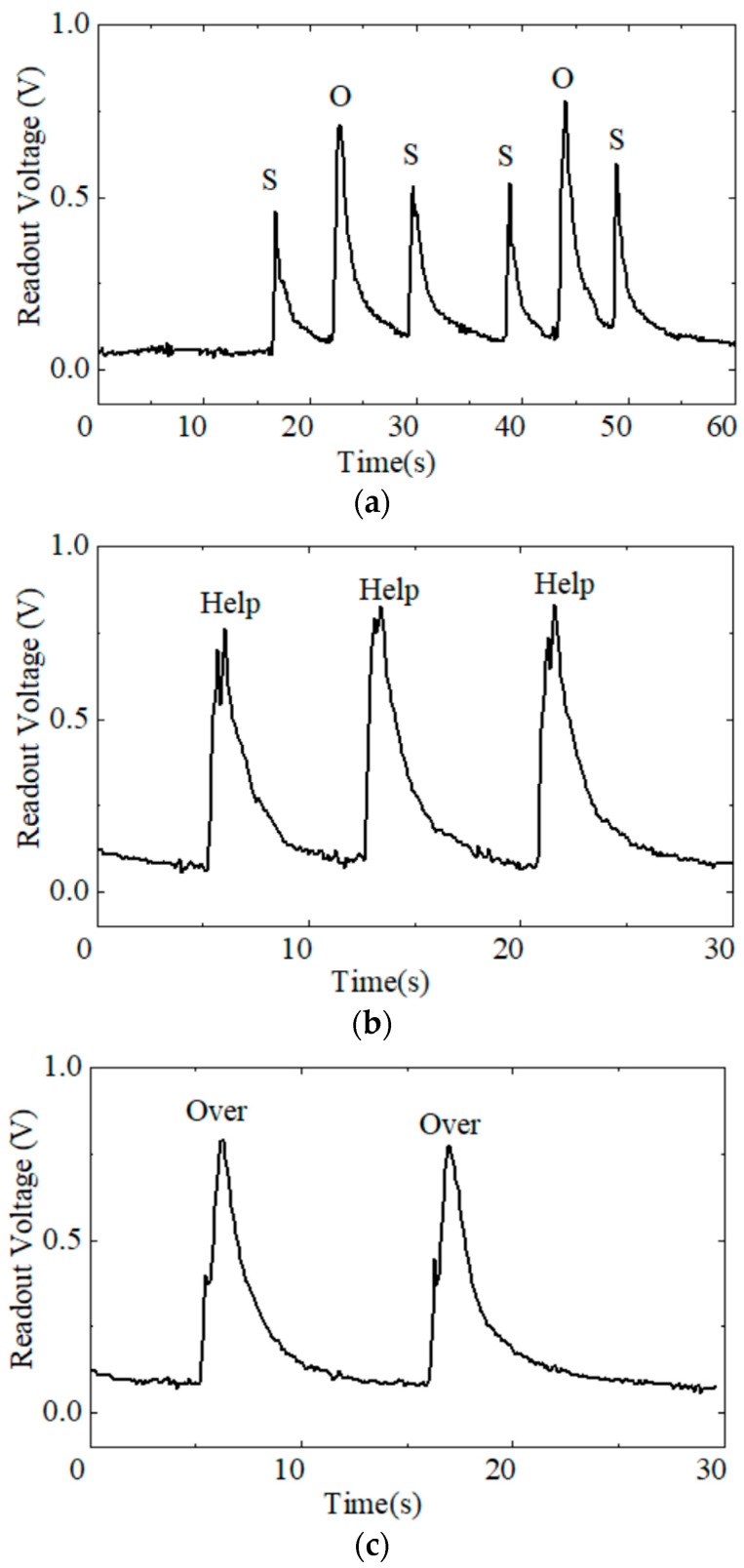
Voltage waveforms of the chitosan humidity sensor for human exhaled air detection during speaking and responses to different characters and words: (**a**) SOS, (**b**) help, and (**c**) over.

Compared with respiratory monitoring, the humidity sensors for speech recognition need faster response and recovery speeds [23]. The air exhaled from the mouth differs when speaking different characters or words. The chitosan-based humidity sensors can sensitively capture water molecules in exhaled air, which leads to changes in the humidity-related readout voltage. There is correspondence between the changing voltage waves and the speaking airflow. For example, the amplitude of the voltage indicates different characters. The peak for “S” was lower than the peak for “O” (Figure 8a) because the sensor detected a larger amount of exhaled air while pronouncing “O” than “S”. It should be stated that the sensors were put away from the mouth in the interval between “O” and “S”. Therefore, there was no peak for breathing during speaking. As our humidity sensors have a fast response rate and a large voltage change range, they can sensitively distinguish many different characters. The waveform is also unique when saying different words. For instance, “help”, as shown in Figure 8b, had a wave shape like an “M”. When speaking “help”, the waveform exhibited a peak during pronouncing/h/, a valley during pronouncing/el/, and a higher peak during pronouncing /p/. However, the wave shape was much different when speaking “over” (Figure 8c). The first peak was short and low. The second peak was wider and higher. Therefore, the various signals carried by the waves can be used to determine the corresponding characteristics of the speaking words.

## 4. Conclusions

This study introduced a simple flexible capacitive humidity sensor, using CS as a humidity-sensitive layer that can be used for wearable respiratory monitoring. The test results show that the sensor capacitance changed from 10^−2^ nF to >30 nF when the environmental humidity changed from 43% to 97%. The logarithm sensitivity was 35.9 pF/%RH in the RH range of 56–97%. The voltage changes read using the Arduino circuit board were nearly 0.8 V from 56% to 97% RH. And the response voltage variation between inhalation and exhalation was ~0.5 V during normal breathing. Our flexible sensors with simple fabrication processes have a good sensing performance. And they can monitor human respiration and distinguish tonal changes well. This low-cost, flexible, highly sensitive humidity sensor provides a scheme for the design of intelligent sensors and intelligent health monitoring systems.

## Data Availability

Data are contained within the article.
